# Looking for Evidence of Public Health's Role for Long-Term Evacuees

**DOI:** 10.3389/fpubh.2019.00015

**Published:** 2019-02-12

**Authors:** Barbara Clow, Margaret Haworth-Brockman, Geneviève Boily-Larouche, Zeeshan Qadar, Yoav Keynan

**Affiliations:** ^1^Barbara Clow Consulting, Halifax, NS, Canada; ^2^National Collaborating Centre for Infectious Diseases, Rady Faculty of Health Sciences, University of Manitoba, Winnipeg, MB, Canada; ^3^Department of Medical Microbiology, Rady Faculty of Health Sciences, University of Manitoba, Winnipeg, MB, Canada

**Keywords:** public health, evacuation, disaster, long-term evacuees, Canada, emergency

## Abstract

Many Canadians have had personal experience of a major emergency or disaster at some point in their lifetime and close to a third of those affected were evacuated from their homes or communities. Most evacuations have lasted less than 2 weeks, but in some instances, people have been displaced for months or years. For example, hundreds of residents evacuated following flooding in Lake St. Martin, Manitoba in 2011, remain displaced today. In order to learn more about the roles and responses of public health for long-term evacuees (LTEs) in Canada, we conducted a narrative review of published English-language documents, beginning with literature specific to Canada and then expanding to include literature on other high-income countries. We found that while researchers have explored public health considerations in emergency preparedness, acute disaster management, and resettlement in these contexts there is a dearth of published evidence regarding the public health implications of prolonged evacuation and the public health responses to long-term evacuation in Canada and in other high-income countries. Because the public health needs of diverse populations of LTEs have not been fully investigated, it is likely that they are neither well-understood nor adequately addressed in public health policy and practice.

## Introduction: Disaster and Evacuation in Canada

In a 2014 survey, 43% of Canadians 15 years and older reported having experienced a major emergency or disaster in their lifetime and close to 30% of those affected have been evacuated from their homes or communities (1). Most of these evacuations last less than 2 weeks, but in some instances, people were displaced for months or years. For example, hundreds of residents evacuated following 2011 flooding in Lake St. Martin, Manitoba remain displaced today (2–5). The full extent of long-term evacuation in Canada is unknown because the Canadian Disaster Database tracks the number of evacuees, but not the duration of displacement. However, anecdotal evidence suggests that individuals in other Canadian communities, such as Lac-Mégantic, Quebec and Fort MacMurray, Alberta, have also experienced prolonged evacuation.

In recent years, communicable disease specialists and public health officers in Manitoba have observed that individuals and families evacuated to Winnipeg from their home communities following disasters, including those from Lake St. Martin, are regularly seeking primary care through emergency departments. This has raised concerns about the role of public health in managing infectious diseases, such as tuberculosis, blood-borne and sexually-transmitted infections, and the on-going public health needs of evacuees who experience extended displacement. The National Collaborating Centre for Infectious Diseases (NCCID), which has a mandate for public health knowledge brokering in Canada, agreed to conduct a review of available literature on current and potential roles for public health during prolonged evacuation.

The following broad research question was formulated:

- “What is known, and what, if anything, is being done about the public health needs of LTEs in Canada?”

## Methods

Reviews are undertaken with the goal of synthesizing evidence available in published literature, but the choice of review method depends upon the nature of the research question (6, 7). Systematic reviews are designed to answer narrowly-defined research questions, often related to clinical practice or outcomes, and they are characterized by strict protocols for identifying, analyzing, and reporting on evidence. Narrative reviews, in contrast, are designed to address broad research questions, such as the state of knowledge about a concept, theory, or context, and to identify trends, patterns, and gaps in literature (8, 9). Because narrative reviews are intended to be iterative, rather than linear, it is difficult to apply strict protocols for searching, selecting, and analyzing evidence (8, 9).

The nature of our research question and the goal of understanding the scope of evidence on public health and long-term evacuation in Canada and other high-income countries determined that a narrative review would be the most appropriate methodology. In keeping with recommendations by Ferrari and others (6, 7, 10) for transparency and rigor, we have described our approach to searching for and selecting relevant literature and to analysis.

We conducted a search for English-language, peer-reviewed literature published between 2012 and 2018. “Long-term” was not defined in these searches to allow for diverse meanings to surface in the literature and to inform the analysis.

A preliminary screening search through Google Scholar was undertaken using search terms related to “public health,” “disaster,” “long-term,” “internally-displaced,” and “Canada.” This search returned more than 20,000 documents, but a rapid scan of abstracts revealed that, in the Canadian context, the term “internally-displaced” was typically associated with individuals who had experienced long-term displacement in their countries of origin, as a result of natural disasters or conflict, before re-settling Canada as immigrants and refugees. It did not refer to those experiencing long-term displacement from homes and communities within Canada. To test this result, we undertook an advanced search in Google Scholar that included the keyword “internally-displaced” and excluded the keywords “immigrant” and/or “refugee^*^” in combination with the other keywords related to public health and disasters in Canada. No relevant documents were identified through this search.

These results led us to adjust our search strategy, replacing keywords related to “internal-displacement” with “long-term evac^*^.” A second screening search in Google Scholar using these keywords returned 731 documents and a rapid scan of abstracts indicated that some had potential relevance to the research question. This second search was repeated using PubMed Central and CINAHL to capture both the biomedical and sociological literature. References were hand-searched to identify relevant studies that fell outside of the search parameters. Additional targeted searches were conducted to identify literature on public health and LTEs from other high-income countries. Together, these searches identified a total of 456 documents with potential relevance.

All abstracts were reviewed, and the literature was analyzed thematically to identify content relevant to the research question as well as patterns and gaps in available evidence.

## Results

Although many documents in this dataset initially appeared relevant to the research question, upon review we found that none specifically or explicitly addressed the public health needs of and/or public health responses to LTEs in Canada. A single study from another high-income country – Japan – provided some evidence of where there may be a need as well as a role for public health among LTEs within Canada (11).

### Where Are the Gaps?

The gaps in the literature on public health and long-term evacuation in Canada and other high-income countries fall into three main categories.

First, some studies broadly consider the long-term repercussions of emergencies and disasters in high-income countries, but they pay little or no attention to the effects of evacuation, prolonged or otherwise. For example, Noy and duPont's 2016 literature review on the long-term economic effects of Hurricane Katrina and the 1930s drought in the United States did not discuss the effect of evacuation or displacement (12). Similarly, a 2012 systematic review by Alderman et al. (9) on the health effects of flooding does not mention evacuation at all.

Second, even when researchers have focused on the detrimental effects of disasters and emergency evacuation on health and safety in Canada or other high-income countries, they have seldom considered the potential effects of prolonged vs. short-term displacement (9, 11, 13–17). For example, several studies of the flooding around Lake St. Martin, Manitoba described myriad adverse effects of displacement on Indigenous communities, but none examined the effects of the duration of evacuation (2–5). Likewise, Martin et al. (18) have explored the severe cultural and social effects of forced displacement on Indigenous communities in Canada, but they have not discussed whether or not prolonged evacuation has had a greater impact than displacements of shorter duration. Interestingly, this gap also existed in the international literature, despite being focused on emergencies such as Hurricane Katrina, and the Fukushima and Chernobyl nuclear disasters, that resulted in communities being evacuated for months or years (11, 13, 14, 17). According to Fussell, studies with evacuees from Hurricane Katrina ended after the first year and duration of evacuation was not treated as a variable of analysis (14). Researchers have noted a similar emphasis on the acute effects of displacement following the Fukushima disaster (11). Diminishing attention to the consequences of evacuation may be related to the costs of longitudinal research, the challenges of tracking evacuees over time, and other factors (11), but it has clearly contributed to a knowledge gap. As Fussell concluded, “much less is known about the in- and out-migrants to New Orleans, and even less is known about the long-term displaced” (14).

Third, research that explicitly considers the role of public health in disasters has tended to focus on emergency preparedness, evacuation decisions and processes, and the management of health during and immediately after a disaster (19–23). As Généreux et al. (24) have noted, “public health response during and after disasters has traditionally focused on protecting populations from chemical, biological, and physical threats, particularly among high-risk groups.” By comparison, few studies have addressed the role of public health in ensuring the on-going health of LTEs. Once again, this finding is surprising because there is ample evidence that vulnerable populations—a public health priority—are likely to experience greater adverse effects from disasters and emergency evacuation (4, 9, 20, 25, 26).

### Where Is There Evidence?

This literature search found a single study that examined the impact of prolonged evacuation following emergencies in a high-income country. Nomura et al. (11) reported on the health of evacuees from the 2011 Fukushima nuclear disaster in Japan. The researchers observed that, 4 years after the disaster, long-term evacuees were more likely than non-evacuees and temporary evacuees to experience weight changes, sleep disturbances, and hyperlipidemia. They noted that risk of hyperlipidemia in LTEs might be related in part to reduced exercise and dietary changes based on real or perceived sustained risk of radiation contamination of air, water, and vegetation, and that public health has an on-going role to play in monitoring and communicating risk (11). They further noted that public health has a responsibility to ensure coordination of and access to services for LTEs.

## Discussion

In recent years, there has been increased interest in and concern about the impact of prolonged and permanent internal displacement on the well-being of individuals, families, and communities. As a result, there is growing body of literature on the physical and mental health effects of internal displacement in low-income countries and fragile or conflict states (27–30), and on the public health needs of IDPs who resettle in Canada and other high-income countries as immigrants and refugees (31, 32). By comparison, little attention has been paid to populations that experience prolonged displacement from homes and communities within Canada or in other high-income countries. This gap may be a function of the reality that a relatively small number of emergencies in high-income countries result in long-term evacuation. In addition, the duration of displacement is difficult to predict at the onset of an emergency. It may also reflect the challenges of defining long-term evacuation and differentiating between the effects of short-term and protracted displacement. The challenges of keeping track of LTEs may also make it difficult to undertake longitudinal research with these populations. But one recent study suggests that those displaced for extended periods following disasters in high-income countries face many challenges and that public health has important roles to play in promoting and protecting the health and safety of LTEs (11).

## Conclusion

Research demonstrates that the likelihood of emergencies associated with climate change is increasing alongside the rising incidence of human-created disasters and humanitarian crises (33, 34). According to the United Nations Office for the Coordination of Humanitarian Affairs, growing numbers of people are being displaced from homes and communities and both crises and displacements are lasting longer (35). Although much of this suffering happens in fragile and conflict states, high-income countries are not immune to disasters or, indeed, to long-term evacuation.

In Canada, emergency preparedness and response fall within the purview of public health (36), which suggests that public health is responsible for the health and safety of LTEs. Given the absence of research in this area, it is difficult to determine if and to what extent the public health effects of prolonged evacuation and the public health needs of long-term evacuees in Canada are being assessed, monitored, and addressed. Trends in the incidence of disasters and emergencies underscore the urgency of conducting more research to improve our understanding of prolonged displacement within Canada and in other high-income countries.

Greater awareness of this evidence gap is critical, both for inspiring new research and for supporting action to address the public health needs of LTEs in Canada. To this end, NCCID will be working with the other National Collaborating Centres for Public Health and public health actors to support knowledge exchange related to the on-going health and safety of LTEs. This will include working toward common definitions of “long-term” as well as how public health roles may continue after immediate and short-term emergency measures have wrapped up. An exploration of the opportunities to use health administrative and other data to augment what is known about the public health needs of diverse populations of LTEs is also proposed. These initial steps will be developed in concert with community leaders and public health officials to advance knowledge translation on environmental, economic, social, and policy implications for populations in Canada displaced long-term from their homes and communities as a result of disasters.

## Limitations

This review considered only English-language published literature.

## Author Contributions

BC was the primary author. MH-B, GB-L, ZQ, and YK conceived the idea of the manuscript and made substantial contributions to all drafts.

### Conflict of Interest Statement

The authors declare that the research was conducted in the absence of any commercial or financial relationships that could be construed as a potential conflict of interest.
